# Supplementary material for: Acute isolation is associated with increased reward seeking and reward learning in human adolescents
Source: Commun Psychol. 2025 Sep 5;3:135. doi: 10.1038/s44271-025-00306-6 (PMC12413320; doi:10.1038/s44271-025-00306-6)
Supplement: Supplementary file 2 — Supplementary Information [file 44271_2025_306_MOESM2_ESM.pdf]

Supplementary Information

Acute isolation is associated with increased reward seeking and reward learning in human adolescents

Livia Tomova[1,2,3]*, Emily Towner[1], Kirsten Thomas[1,4], Lei Zhang[5,6,7], Stefano Palminteri[8,9], Sarah-Jayne Blakemore[1,4]

[1] Department of Psychology, University of Cambridge, Cambridge, UK
[2] Cardiff University Brain Research Imaging Centre (CUBRIC), Cardiff University, Cardiff, UK
[3] Cardiff University Centre for Human Developmental Science (CUCHDS), Cardiff University, Cardiff, UK
[4] Institute of Cognitive Neuroscience, University College London, London, UK
[5] Centre for Human Brain Health, School of Psychology, University of Birmingham, Birmingham, UK
[6] Institute for Mental Health, School of Psychology, University of Birmingham, Birmingham, UK
[7] Centre for Developmental Science, School of Psychology, University of Birmingham, Birmingham, UK
[8] Departement d'Etudes Cognitive, Ecole Normale Superieure, Paris, France
[9] Laboratoire de Neurosciences Cognitives et Computationnelles, Institut National de la Santé et de la Recherche Médicale, Paris, France

*Corresponding authors: tomoval@cardiff.ac.uk, sjblakemore@psychol.cam.ac.uk

Supplementary Notes

<u>Supplementary Note 1: Analyses for final rating at the end of isolation (T4; 3h 30min to 4h of isolation, varying between participants)</u>

At the end of social isolation (T4), participants reported significantly increased loneliness compared with at the start of the session, in both isolation sessions (iso total: $t(38)=5.88$, $P< 0.001$, Cohen's $d=0.99$; iso with media: $t(39) =4.92$, $P<0.001$, Cohen's $d=0.71$) and decreased positive mood (iso total: $t(38)=-6.18$, $P<0.001$, Cohen's $d=0.79$; iso with media: ($t(39)=-6.31$, $P<0.001$, Cohen's $d=0.76$). Supplementary Figure 3 shows plots including T4 measures.

There was no change in social craving over the duration of isolation (for iso total and iso with media) at T4 or T3 compared to the start of the session in either session (iso total: T0 vs. T3: $t(39) =0.65$, $P=0.518$; T0 vs. T4: $t(39) =0.15$, $P=0.884$; iso media: T0 vs. T3: $t(39) =0.68$, $P=0.501$; T0 vs. T4: $t(39) =-0.12$, $P=0.909$; see Figure 2 in the main text for T0-T3 data).

<u>Supplementary Note 2: Effects of session on loneliness, social craving, state anxiety, boredom, positive mood and negative mood</u>

Analyses of social craving, boredom and negative mood were conducted on measures taken after 3 hours (T3) of isolation as described in the main text (see methods section for details).

We compared effects between all three sessions (baseline, iso total and iso with media) comparing measures taken at T3 for the two isolation sessions to the baseline measure. To do this, we included session (baseline, iso total and iso with media) as a predictor in a mixed effects model to test for differences between sessions on self-reported state measures with subject included as random effect (with random intercepts). Iso total was the reference session in this model.

The command was: fitlme(Data,'outcome~(session + (1 |subjectID)').

We ran six separate models to test for effects on loneliness, social craving, state anxiety, boredom, positive mood and negative mood and report results as significant at $p < 0.008$ (0.05/6).

Loneliness

Participants self-reported higher loneliness in the iso total session compared to baseline ($b=-32.45$, $t(117)=-7.25$, CI=-41.31,-23.59, $P<0.001$) and compared to the iso with media session ($b=-12.73$, $t(117)=-2.84$, CI=-21.59,-3.86, $P=0.005$).

Social Craving. Participants self-reported lower social craving in the iso total session compared to baseline (b=19.88, t(116)=4.96, CI=11.94, 27.81, P<0.001) but not compared to the iso with media session (b=4.13, t(116)=1.02, CI=-3.87,12.13, P=0.309).
State Anxiety. There was no effect of session on state anxiety (baseline: b=-1.17, t(116)=-0.88, CI=-3.81, 1.47, P=0.382; iso with media: b=-0.87, t(116)=-0.65, CI=-3.51,1.77, P=0.516).
Boredom. Participants self-reported higher boredom in the iso total session compared to baseline (b=-34.84, t(116)=-7.60, CI=-43.92,-25.76, P<0.001) and compared to the iso with media session (b=-19.49, t(116)=-4.25, CI=-28.57,-10.41, P<0.001).
Positive Mood. Participants also self-reported less positive mood in the iso total session compared to baseline (b=10.24, t(115)=8.77, CI=7.93, 12.55, P<0.001) but not compared to the iso with media session (b=1.34, t(115)=1.15, CI=-0.97,3.65, P=0.254).
Negative Mood. Participants also showed a trend towards lower self-reported negative mood in the iso total session compared to baseline, but this effect did not survive correction for multiple comparisons (b=1.26, t(115)=2.56, CI=0.01, 0.29, P=0.012). There was also no effect compared to the iso with media session (b=-0.37, t(115)=-0.75, CI=-1.34,0.60, P=0.453).

<u>Supplementary Note 3: Sensitivity analysis reward seeking: Effects of session order</u>

There were three sessions (baseline, iso total and iso with media), and the order of the iso total and iso with media session was counterbalanced between sessions 2 and 3 across participants. Thus, iso total was session 2 for half the participants and session 3 for the other half (and vice versa for iso with media). To test if session order could be driving the effects of isolation on reward seeking, we added session order (iso total first, iso with media first) as an additional predictor in our linear mixed effects model. We specified this model with random intercepts but without random slopes as we were not able to accomplish a fully random model with 5 interaction terms (i.e. the model did not converge, and we therefore reduced complexity by removing random slopes).
The command was: fitlme(Data,'outcome~(session*reward * effort* context* session_order) + (1 |subjectID)').

We did not find a main effect of session_order (b=-0.16, t(708)=-1.25, CI=-0.40,0.09, P=0.211) or an interaction between session and session_order and either session (baseline: b=-0.04, t(708)=-0.26, CI=-0.29,0.22, P=0.792; iso with media: b=-0.10, t(708)=-0.78, CI=-0.37,0.16, P=0.435) .

Supplementary Note 4: Sensitivity analysis: Isolation-induced changes in positive mood and boredom and changes in social reward seeking

We calculated a sensitivity analysis testing if the association between changes in loneliness and changes in reward seeking reported in the main text was specific to loneliness or whether we would find a similar association with other state variables which showed significant changes during isolation (i.e., boredom and positive mood). Analogous to the analysis reported in the main text, we used RTs in high reward, high effort trials, assessed separately for each context (social, nonsocial) and report results as significant at Bonferroni corrected p<0.025 (0.05/2).

Boredom. We found no correlation between difference in boredom and difference in RTs in the social context (r(38)=-0.261; p=0.109, CI=-0.52,0.07) or the non-social context (r(38)=-0.062; p=0.709, CI=-0.36,0.26).

Positive mood. We found no correlation between difference in positive mood and difference in RTs in the social context (r(38)=0.067; p=0.650, CI=-0.21,0.41) or the non-social context (r(38)=-0.364; p=0.025, CI=-0.48,0.41).

Supplementary Note 5: Sensitivity analysis reward learning: Effects of session order

RTs

Analogous to above, to test if session order could be driving the effects of isolation on RTs during reward learning we added session order (iso total first, iso with media first) as an additional predictor in our linear mixed effects model. We specified this model with random intercepts and random slopes, as in the original model.

The command was: fitlme(Data,'outcome~(session*feedback*phase*session_order) + (session*feedback*phase*session_order |subjectID)').

We did not find a main effect of session_order (b=0.14, t(936)=0.98, CI=-0.14,0.42, P=0.329) or an interaction between session and session_order and either session (baseline: b=-0.15, t(936)=-0.78, CI=-0.52,0.22, P=0.436; iso with media: b=-0.12, t(936)=-0.58, CI=-0.51,0.28, P=0.564).

Reward Learning Model

To explore whether session order might have impacted effects on parameters from the modelling approach, we tested another model, which was identical to the original winning model (i.e., the fictitious update model) but explicitly included session order as an additional predictor to test if this model showed a better fit to the data. The model did not show a better fit (WAIC new model = 6363; WAIC original winning model = 6315.901 (Supplementary Table 4)). Thus, adding information about session order did not improve model fit, suggesting that order was not able to explain the variance in the data structure and was not driving the effects.

<u>Supplementary Note 6: Sensitivity analysis: Isolation-induced changes in positive mood and boredom and changes in learning_neg rates.</u>

We calculated a sensitivity analysis testing if the association between changes in loneliness and changes in learning_neg rates reported in the main text was specific to loneliness or whether we would find a similar association with other state variables which showed significant changes during isolation (i.e., boredom and positive mood). Analogous to the analysis reported in the main text, we ran this analysis separately for each context (social, nonsocial) and report results as significant at Bonferroni corrected $p<0.025$ (0.05/2).

Boredom. We found no correlation between difference in boredom and difference in RTs in the social context ($r(38)=0.347$; $p=0.030$, CI=0.04,0.60) or the non-social context ($r(38)=0.271$; $p=0.095$, CI=-0.05,0.54).

Positive mood. We found no correlation between difference in positive mood and difference in RTs in the social context ($r(38)=0.082$; $p=0.624$, CI=-0.22,0.41) or the non-social context ($r(38)=0.053$; $p=0.755$, CI=-0.23,0.39).

Supplementary Note 7: Planned analyses and deviations from preregistration

Given that our preregistration involved a larger project (osf.io/w5um9), which included different measures that are not all reported in the current manuscript, we created a detailed list of all hypotheses (Part 1) that are addressed in the present manuscript. In addition, below we include a detailed description on whether and how the current analyses deviated from our original analysis plan (Part 2) published in the preregistration.

Part 1: Hypotheses

Hypotheses addressed in the present paper

H1) Emotions: Isolation will increase negative mood and decrease positive mood; increase loneliness and social craving and increase anxiety; all measured by self-report ratings.

H3) Reward responsiveness: Isolation will increase the willingness to expend effort to obtain monetary rewards, as measured by an effort-based decision-making task. Further, responsiveness to monetary efforts will be higher in a social context (in the social condition, in addition to obtaining monetary rewards, participants will see social pictures) compared to a non-social context (in the non-social condition, in addition to obtaining monetary rewards, participants will see landscape pictures) following isolation, and both will be increased compared to baseline.

H4) Reward learning: Isolation will alter reward learning, as measured by a reinforcement/reversal learning task. Isolation will enhance reinforcement learning, but diminish reversal learning, i.e., participants will initially learn associations between cues and rewards faster but will show perseveration. These effects (faster learning but higher perseverance) will be stronger for social compared to monetary rewards.

H7) Social media use during isolation will remediate the effects of isolation on emotions, cognitive control, reward responsiveness, reward learning, fear learning and susceptibility to peer influence on risk perception.

H8) Participants who show higher neural sensitivity to rewards will be more sensitive to the effects of isolation, especially in outcome measures related to reward processing (i.e., reward responsiveness and reward learning).

Part 2: Analysis plan

Below, we include the analyses addressed in the current work and report whether and how we deviated from the preregistered analysis plan. We are not including preregistered analyses we did not implement in the current work here (these analyses are partially still pending and will be published elsewhere).

Questionnaire Data Analysis

Preregistration:

Emotions and Mood. We will assess how acute isolation affects participants' state anxiety (STAI state), positive and negative mood (PANAS), and social craving/acute loneliness (Acute Loneliness Questionnaire).

Substance Craving. We will assess how isolation affects participants' self-reported desire to consume substances.

For each of these measures separately, we will use mixed effects models to test for differences between sessions (baseline, iso_total, and iso_media) on state anxiety, positive and negative mood, social craving/ loneliness and substance craving, with subject included as a random effect.

Actual analysis implemented:

We analysed all variables as preregistered (analyses reported in the SI) except for the substance craving variables because our sample reported very low substance use of the variables we assessed (only 55% (n=22) reported that they drank alcohol at all and only 2 participants reported that they vaped). As reported in the methods section on Questionnaires, we did not consider this to be a large enough sample to have sufficient power to assess effects of isolation on substance use and dropped these variables from the analysis.

We deviated from the preregistered analysis by adding an exploratory analysis testing if self-report measures changed throughout the isolation sessions. To do this, we first compared measures taken at T0 to those taken at T3 using paired sample t-tests. Second, to assess if the change over time (i.e. the slope) was different between the two isolation sessions, we used linear mixed-effects models with the following predictors: session (iso total and iso with media) and duration (hours of isolation: 0, 1, 2, 3) with subject included as a random effect. Note that we did not include the

baseline session in this comparison as this session did not have the relevant measures (i.e., repeated collection of state measures throughout isolation).

The command was: fitlme(Data,'self-report measure~(session*duration)+ (session*duration|subjectID)').

We included these exploratory analyses in the main text because we think they better capture the effects of isolation on these measures compared to the analysis we preregistered.

<u>Preregistration:</u>

We will also use our measures of chronic loneliness (UCLA loneliness scale), trait anxiety (STAI trait), trait depression (CES-D), and social network size (social network scale) as predictors of state anxiety (STAI state) negative mood (PANAS) and social craving (social craving questionnaire) following acute isolation. These predictors are truncated as we will exclude participants with high chronic loneliness, low social network size and/or diagnosed anxiety or depression during eligibility screening. We will therefore use a truncated regression model when running these prediction analyses.

<u>Actual analysis implemented:</u>

We did not analyse whether chronic loneliness (UCLA loneliness scale), trait anxiety (STAI trait), trait depression (CES-D), or social network size (social network scale) predicted state anxiety, negative mood and social craving following isolation as originally planned, because we did not find any effects of isolation on state anxiety, negative mood and social craving.

Behavioural Data Analysis

<u>Preregistration:</u>

Reward Responsiveness. Using data from the effort-based decision-making task, we will calculate the sum of number of played trials and mean response times across all trials for the following combinations in each condition (non-social or social): high effort - high reward; low effort – high reward; high effort – low reward; low effort - low reward (8 conditions in total). We will assess whether the number of played trials and response times for deciding to play differs between sessions (baseline, iso_total, and iso_media). We will also generate means across the different conditions (non-social and social) and test for differences between sessions for each combination

(across both non-social and social): high effort - high reward; low effort – high reward; high effort – low reward; low effort-low reward. We will use mixed effects models to test for differences between sessions to estimate the fixed effects of effort (high, low), reward (high, low) and session (baseline, iso_total, and iso_media) in each context (non-social, social) on error rates and response times, with subject included as a random effect.

Actual analysis implemented:

Because we considered the term "reward responsiveness" not to capture accurately the construct measure here, we re-labelled reward responsiveness as reward seeking and used reward responsiveness as an umbrella term incorporating reward seeking and reward learning in the present paper. Thus, the hypotheses on reward responsiveness are directed at reward seeking. We analysed this data as preregistered. We noticed a typo in the preregistration, as the final sentence states that we will analyse "error rates and response times" but it should be "number of trials played and response times" as there are no error rates in this task.

As reported in the methods section, because of the low variance in participants' choice data (a substantial number of participants chose to play every trial in the task), we decided to use response times (RTs) as the main measure of reward seeking but dropped the analysis of choice data.

Preregistration:

Reward Learning. Participants' choices from the reinforcement/reversal learning task will be analysed using a computational reinforcement learning and decision-making model for probabilistic reversal learning tasks (Ahn 2017, Metha 2020). We will estimate the following parameters for each participant: learning rate, experience decay factor and inverse temperature. The learning rate is based on how much an individual updates their current response based on their most recent response, the experience decay factor is a measure of perseveration, and inverse temperature is a measure of exploration versus exploitation (den Ouden 2013).
To compare differences in these estimates, we will use mixed effects models to test for differences between sessions in order to estimate the fixed effects of feedback condition (social versus non-

social) and session (baseline, iso_total, and iso_media) on our three parameters of interest (learning rate, experience decay factor and inverse temperature), with subject included as a random effect.

<u>Actual analysis implemented:</u>

We analysed this data as preregistered but found that the model including the experience decay factor (a perseverance factor), which we initially thought would fit the data best due to the predicted perseveration effects (see hypothesis H3 in the main text), was not the best fit for the data. We instead found that the fictitious update model, which uses separate learning rates for positive and negative prediction errors (learning_pos, learning_neg) showed better fit to the data. We therefore chose to analyse data using the fictitious update model. As this model did not include a perseverance parameter, we did not run follow-up analyses testing for differences in this parameter. We ran the preregistered analyses for learning rates and inverse temperature. We measured perseveration (and general task performance) using behavioural performance measures from the task. Specifically, we assessed perseverative errors, which we quantified as the proportion of errors choosing the previously reinforced stimulus following each reversal. (see methods section in main text for details). We also assessed accuracy, which was quantified as the proportion of correct responses. In addition, we assessed response times (RTs) for making choices during the task. Because our version of the task included a time limit for responding (1s), hence making fast responding advantageous, we also assessed response times for making choices during each phase of the task.

We also did not compare participant-level model parameters between sessions using frequentist analyses as originally planned, because we realised that such two-step approaches combining hierarchical Bayesian models with frequentist follow-up analyses were shown to systematically underestimate variance resulting in a bias towards the alternative hypothesis (Boehm 2018). Instead, we use Bayesian hypothesis testing as recommended by Boehm 2018.

Supplementary Figures

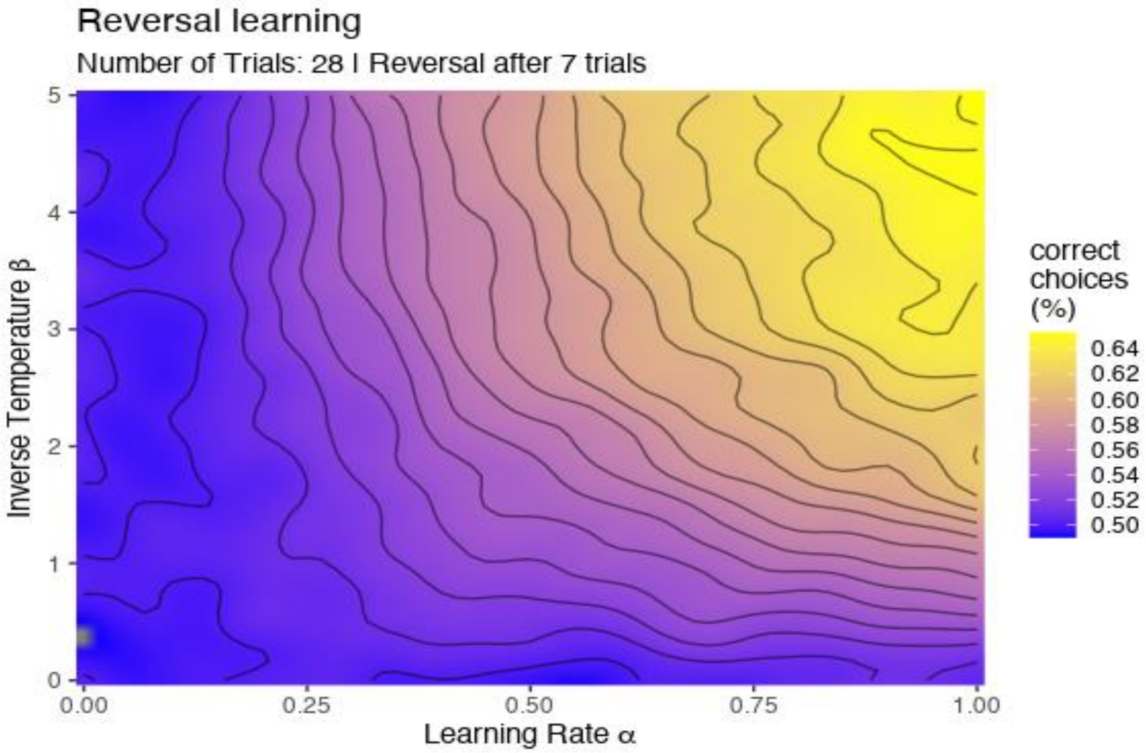

Supplementary Figure 1. Simulations of optimal parameters RL task.
The figure shows the optimal combination of learning rate and inverse temperature predicting highest choice accuracy for the task setup used in this study. The values are obtained from data simulations based on the assigned reward probabilities, task length and frequency of reversals. Bright yellow colouring depicts high choice accuracy while purple colouring indicates low choice accuracy (50% equals chance performance). The yellow area indicates the optimal combination of learning rate and inverse temperature predicting the highest choice accuracy for the present task setup.

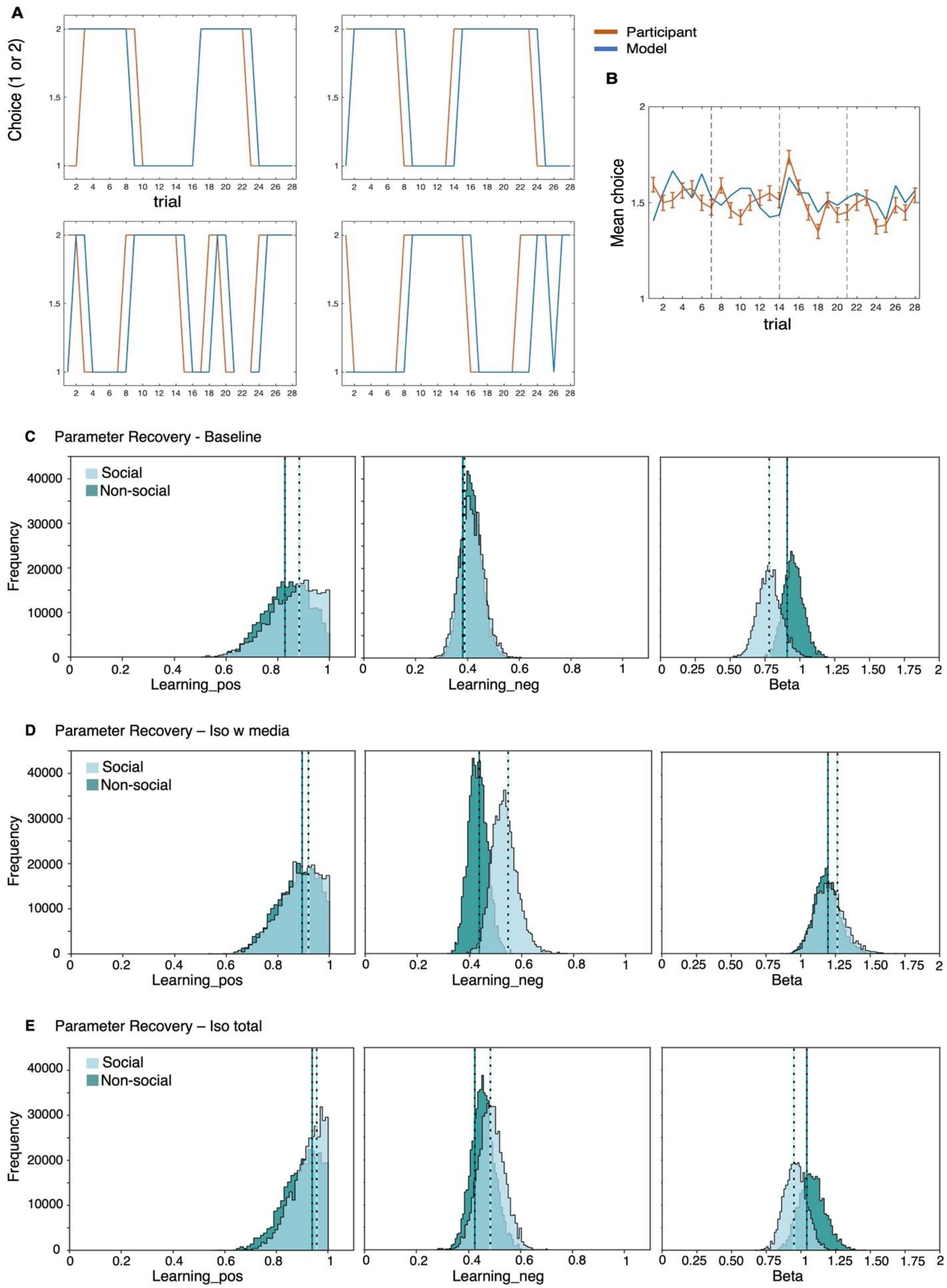
A
Choice (1 or 2)
trial
Participant
Model
B
Mean choice
trial
C Parameter Recovery - Baseline
Social
Non-social
Frequency
Learning_pos
Learning_neg
Beta
D Parameter Recovery – Iso w media
Social
Non-social
Frequency
Learning_pos
Learning_neg
Beta
E Parameter Recovery – Iso total
Social
Non-social
Frequency
Learning_pos
Learning_neg
Beta

Supplementary Figure 2. Posterior predictive checks and parameter recovery RL task. **A.** True data and simulated choice estimate data from the fictitious update model for four exemplar participants. Red lines represent the true data, and blue lines represent the simulated data from the model. **B.** Mean participant choice across all participants (+- SEM; in red) and mean simulated choice estimates (in blue) for each trial. The dotted lines indicate the reversals of reward contingencies (i.e., every 7 trials). The plots illustrate how well the model captures the pattern seen in the data. **C-E**. Parameter recovery. We extracted the mean and standard deviation for each group-level posterior distributions for each parameter (learning_pos, learning_neg and beta) as the "ground truth" and generated n = 40 individual level parameters for each case (i.e., each combination of session (baseline, iso with media and iso total) and feedback (social, non-social)). Simulated data with those individual level parameters was generated from the fictitious update model and the model was then fit to the simulated data. The posterior group level distributions from each recovered parameter for each case was compared to the ground truth. Parameters are considered well recovered if the ground truth is within the 95% highest density interval (HDI) of the posterior distribution which was the case for each parameter. Parameter recovery was therefore successful for all parameters. The histograms depict the posterior distributions for the recovered parameters compared against the ground truth for that parameter, which is depicted as the dashed line for the social feedback (light blue) and the non-social feedback (green).

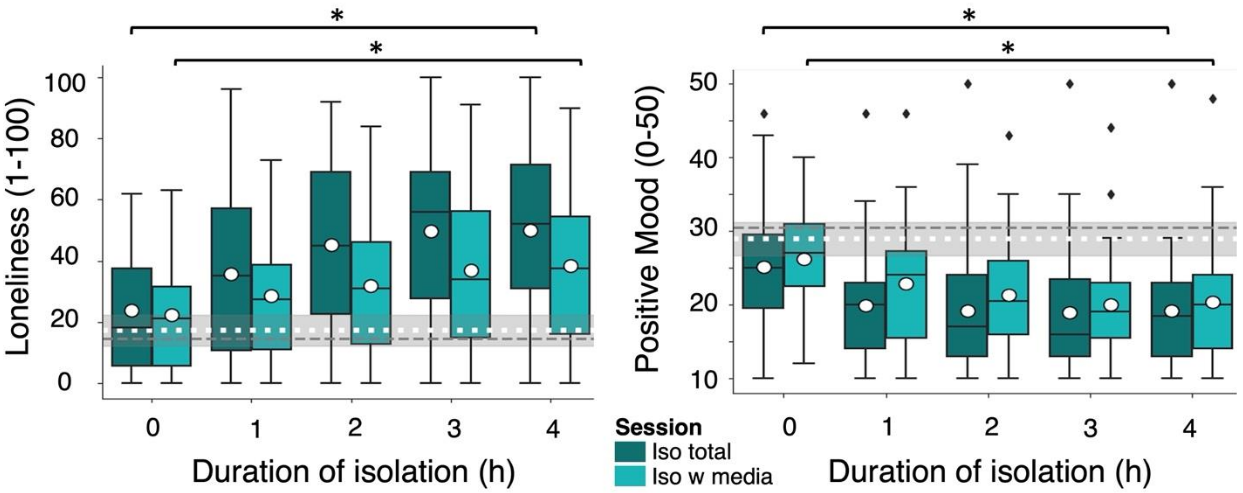

Supplementary Figure 3. Effects of isolation on loneliness and mood including T4. Changes in self-reported loneliness (left) and positive mood (right) over time during isolation. Dark green = iso total session; turquoise = iso with media session. The boxplots indicate the median (black centre line), mean (white circle), the interquartile range (IQR; box) and the 1.5 x IQR minima and maxima (whiskers). The black rhombi indicate outliers (values outside of 1.5 x IQR). The dashed lines across the plots indicate the mean (white) and median (grey) ratings during baseline and their 95% confidence interval (grey area). * indicates $p < 0.001$.

Supplementary Tables

Supplementary Table 1. Full output linear mixed effects model for EBDM task.

**Predictors**	**Estimates**	**t (df)**	**p**
Session: Baseline	0.315	5.06 (732)	<0.001
Session: Iso with media	0.159	2.55 (732)	0.011
Context: Social	-0.051	-1.07 (732)	0.285
Reward: High	-0.035	-0.65 (732)	0.519
Effort: High	0.031	0.66 (732)	0.512
Interaction: Session (Baseline) – Context (Social) – Reward (High)	0.076	-0.70 (732)	0.487
Interaction: Session (Iso with media) – Context (Social) – Reward (High)	-0.262	-3.0 (732)	0.003

Supplementary Table 2. Results of model comparison for RL task.

Model	LOOIC	Bayesian stacking	Pseudo-BMA	WAIC
RP	6496.026	0.137	0.033	6420.713
EWA	6547.515	0.000	0.000	6445.719
EWArp	6510.420	0.330	0.025	6418.882
FU	6388.746	0.533	0.942	6315.901

We report the following model comparison metrics: LOOIC = Leave-One-Out Information Criterion; Bayesian Stacking = stacking of means[1]; Pseudo-BMA = Pseudo Bayesian Model Averaging[1]; WAIC = Widely Applicable Information Criterion; RP = Reward-Punishment Model; EWA = Experience-Weighted Attraction Model; EWArp = Experience-Weighted Attratction Model with separate learning rates for positive and negative prediction errors; FU = Fictitious Update Model. For LOOIC and WAIC, lower values indicate better model fit. For Stacking and Pseudo-BMA higher values indicate better model fit. We used Bayesian Stacking and Pseudo-BMA as model weight metrics to determine the winning model (see methods for details). The Fictitious Update Model is marked grey as it shows the best model fit.

[1]Yao, Y., Vehtari, A., Simpson, D. & Gelman, A. Using Stacking to Average Bayesian Predictive Distributions (with Discussion). Bayesian Anal. 13, 917–1007 (2018).
