# Supplementary material for: Acute isolation is associated with increased reward seeking and reward learning in human adolescents
Source: Commun Psychol. 2025 Sep 5;3:135. doi: 10.1038/s44271-025-00306-6 (PMC12413320; doi:10.1038/s44271-025-00306-6)
Supplement: Supplementary file 3 — Reporting-Summary [file 44271_2025_306_MOESM3_ESM.pdf]

nature portfolio

Corresponding author(s): Livia Tomova

Last updated by author(s): Jul 22, 2025

Reporting Summary

Nature Portfolio wishes to improve the reproducibility of the work that we publish. This form provides structure for consistency and transparency in reporting. For further information on Nature Portfolio policies, see our Editorial Policies and the Editorial Policy Checklist.

Statistics

For all statistical analyses, confirm that the following items are present in the figure legend, table legend, main text, or Methods section.

n/a	Confirmed	
☐	☒	The exact sample size (*n*) for each experimental group/condition, given as a discrete number and unit of measurement
☐	☒	A statement on whether measurements were taken from distinct samples or whether the same sample was measured repeatedly
☐	☒	The statistical test(s) used AND whether they are one- or two-sided<br>*Only common tests should be described solely by name; describe more complex techniques in the Methods section.*
☐	☒	A description of all covariates tested
☐	☒	A description of any assumptions or corrections, such as tests of normality and adjustment for multiple comparisons
☐	☒	A full description of the statistical parameters including central tendency (e.g. means) or other basic estimates (e.g. regression coefficient) AND variation (e.g. standard deviation) or associated estimates of uncertainty (e.g. confidence intervals)
☐	☒	For null hypothesis testing, the test statistic (e.g. *F*, *t*, *r*) with confidence intervals, effect sizes, degrees of freedom and *P* value noted<br>*Give P values as exact values whenever suitable.*
☐	☒	For Bayesian analysis, information on the choice of priors and Markov chain Monte Carlo settings
☐	☒	For hierarchical and complex designs, identification of the appropriate level for tests and full reporting of outcomes
☐	☒	Estimates of effect sizes (e.g. Cohen's *d*, Pearson's *r*), indicating how they were calculated

Our web collection on statistics for biologists contains articles on many of the points above.

Software and code

Policy information about availability of computer code

Data collection	Behavioural data reward tasks: PsychoPy (code for tasks available at: https://osf.io/qrhd6/); Questionnaire data: REDCap; Qualtrics
Data analysis	Behavioural tasks: Python Jupyter Notebook for data processing (code available at: https://osf.io/qrhd6/); for Reward Learning task: fitting of reinforcement learning models done via hBayesDM library in RStudio (code available at: https://osf.io/qrhd6/); Matlab's fitlme function for statistical analysis (commands used reported in manuscript).

For manuscripts utilizing custom algorithms or software that are central to the research but not yet described in published literature, software must be made available to editors and reviewers. We strongly encourage code deposition in a community repository (e.g. GitHub). See the Nature Portfolio guidelines for submitting code & software for further information.

Data

Policy information about availability of data

All manuscripts must include a data availability statement. This statement should provide the following information, where applicable:

- Accession codes, unique identifiers, or web links for publicly available datasets
- A description of any restrictions on data availability
- For clinical datasets or third party data, please ensure that the statement adheres to our policy

De-identified behavioural data and questionnaire data will be made publicly available on the open science framework before publication.

Human research participants

Policy information about studies involving human research participants and Sex and Gender in Research.

Reporting on sex and gender	We collected information on sex and gender of participants via self-report collected in an online questionnaire. Participants were shown a brief explanation of the terms sex and gender and were asked to provide information on both.<br>In our sample all adolescents described their gender as matching their biological sex.
Population characteristics	Participants (n = 40) were healthy adolescents, ranging in age from 16-19 years (mean age = 17.12 years, standard deviation = 0.90; n = 22 female).
Recruitment	Participants were recruited via online advertisements and flyers. Interested individuals filled out a screening questionnaire to assess eligibility for the study. Because this study was part of a larger project which involved an MRI scan, people were eligible if they reported no permanently implanted metal in their body, no history of brain damage, and no currently diagnosed mental health disorder or substance abuse. Because data collection occurred during the COVID-19 pandemic, other eligibility criteria included: no current COVID positive test, no chronic illness (including asthma), no smoking. Because we aimed to study effects of isolation in a sample of adolescents who have frequent and regular social interactions, we also excluded people who i) lived alone, ii) reported current feelings of loneliness on the UCLA Loneliness Scale (i.e., we excluded people with scores above 50, which is one standard deviation above the mean for a student sample); or iii) reported smaller social network sizes than typically expected of adults according to a social network size measure and the Social Support Questionnaire (i.e., we excluded people with social networks 2 or more SD below mean, based on prior measured distributions from Von der Heide et al. 2014 (see methods section in the manuscript for references)).<br>Participants were primarily adolescents from local schools around Cambridge or first year undergraduate students at Cambridge University.
Ethics oversight	All experimental procedures were approved by the ethics committee of the Department of Psychology at University of Cambridge.

Note that full information on the approval of the study protocol must also be provided in the manuscript.

Field-specific reporting

Please select the one below that is the best fit for your research. If you are not sure, read the appropriate sections before making your selection.

☐ Life sciences ☒ Behavioural & social sciences ☐ Ecological, evolutionary & environmental sciences

For a reference copy of the document with all sections, see nature.com/documents/nr-reporting-summary-flat.pdf

Behavioural & social sciences study design

All studies must disclose on these points even when the disclosure is negative.

Study description	Experimental quantitative within-subjects study design. Each participant underwent three experimental sessions: baseline, total isolation (iso total), and isolation with social media (iso media). We collected behavioural measures during the baseline session which were repeated on the iso total and iso media sessions. This allowed us to compare behavioural effects between sessions (baseline vs. iso total; baseline vs. iso media), the difference in change between the two isolation conditions ((baseline vs. iso total) vs (baseline vs. iso media)).
Research sample	Participants (n = 40) were healthy adolescents, ranging in age from 16-19 years (mean age = 17.12 years, standard deviation = 0.90; n = 22 female). We collected data from 42 participants; 2 participants were unable to complete all experimental sessions and so were dropped from analysis, leaving 40 complete datasets. Participants were primarily adolescents from local schools around Cambridge, UK or first year undergraduate students at Cambridge University.
Sampling strategy	Convenience sample. Pilot data (N = 19; taken from part of the sample in Tomova et al. 2020) from 18–24-year-olds has shown that short-term isolation affects feelings of loneliness (using a self-report loneliness scale ranging from 0-100) after just four hours of isolation with an effect size (Cohen's d) of 0.47. A power analysis showed that 38 participants are required to detect a medium effect size of d = 0.47 in our outcome measures to achieve a power of .80 (1-beta) at an alpha of .05. Recruitment was stopped after 40 participants successfully completed all three sessions of the study. New participants were recruited to account for participants who failed to complete all three sessions (2 participants).
Data collection	Behavioural data reward tasks: PsychoPy (code for tasks available at: https://osf.io/qrhd6/); Questionnaire data: REDCap; Qualtrics<br>Data collection was not performed blind to the conditions of the experiments.
Timing	Data collection started April 2021 and was finished February 2022.
Data exclusions	Exclusion criteria were pre-established in our pre-registration (https://osf.io/w5um9/).

Data exclusions	For the behavioural analysis, trials in which participants did not respond will be not included in the analysis.<br><br>Because we were mainly interested in the relationship between the different measures within each participant (i.e., differences and commonalities between the different sessions (baseline, iso total, iso with media)) and not mean values across the sample, we did not exclude any participants if they deviated from the group mean on any measure.
Non-participation	2 participants stopped participating in the study before finishing all 3 experimental sessions.
Randomization	All participants underwent all the three experimental sessions (baseline, iso total and iso with media).

Reporting for specific materials, systems and methods

We require information from authors about some types of materials, experimental systems and methods used in many studies. Here, indicate whether each material, system or method listed is relevant to your study. If you are not sure if a list item applies to your research, read the appropriate section before selecting a response.

Materials & experimental systems

n/a	Involved in the study
☒	☐ Antibodies
☒	☐ Eukaryotic cell lines
☒	☐ Palaeontology and archaeology
☒	☐ Animals and other organisms
☒	☐ Clinical data
☒	☐ Dual use research of concern

Methods

n/a	Involved in the study
☒	☐ ChIP-seq
☒	☐ Flow cytometry
☒	☐ MRI-based neuroimaging
